# The tardigrade protein CAHS D interacts with, but does not retain, water in hydrated and desiccated systems

**DOI:** 10.1038/s41598-023-37485-3

**Published:** 2023-06-27

**Authors:** Silvia Sanchez-Martinez, John F. Ramirez, Emma K. Meese, Charles A. Childs, Thomas C. Boothby

**Affiliations:** Department of Molecular Biology, University of Wyoming, Laramie, WY 82071 USA

**Keywords:** Biochemistry, Physiology

## Abstract

Tardigrades are a group of microscopic animals renowned for their ability to survive near complete desiccation. A family of proteins, unique to tardigrades, called Cytoplasmic Abundant Heat Soluble (CAHS) proteins are necessary to mediate robust desiccation tolerance in these animals. However, the mechanism(s) by which CAHS proteins help to protect tardigrades during water-loss have not been fully elucidated. Here we use thermogravimetric analysis to empirically test the proposed hypothesis that tardigrade CAHS proteins, due to their propensity to form hydrogels, help to retain water during desiccation. We find that regardless of its gelled state, both in vitro and in vivo, a model CAHS protein (CAHS D) retains no more water than common proteins and control cells in the dry state. However, we find that while CAHS D proteins do not increase the total amount of water retained in a dry system, they interact with the small amount of water that does remain. Our study indicates that desiccation tolerance mediated by CAHS D cannot be simply ascribed to water retention and instead implicates its ability to interact more tightly with residual water as a possible mechanism underlying its protective capacity. These results advance our fundamental understanding of tardigrade desiccation tolerance which could provide potential avenues for new technologies to aid in the storage of dry shelf-stable pharmaceuticals and the generation of stress tolerant crops to ensure food security in the face of global climate change.

## Introduction

Ever since the father of microscopy, Antonie van Leeuwenhoek observed tiny desiccated “animalcules” reanimating after the addition of water to dirt he had dried over the course of a summer, understanding how certain animals are able to cope with losing the hydrating water inside their bodies and cells has fascinated scientists. Despite centuries passing, we still only know of four phyla within the Kingdom Animalia containing species that can perform this trick, known as anhydrobiosis (Greek for “life without water”), including some arthropods, nematodes, bdelloid rotifers, and tardigrades^1–9^. A mechanistic understanding of how these diminutive, but robust, animals survive the extreme stress of desiccation is one of the enduring mysteries of organismal physiology.

Classically, successful anhydrobiosis has been attributed to the accumulation of high levels (~ 20 percent dry mass) of non-reducing disaccharides, such as trehalose^2,3,6,7,10,11^. Trehalose is thought to work to protect organisms, their cells, and cellular components through several protective mechanisms including vitrification, water replacement, stabilization of sensitive proteins via reduced preferential binding to their unfolded state, and as a synergistic cosolute^7,12–17^. Interestingly, despite trehalose being a bonafide mediator of desiccation tolerance, it appears to be made in low levels, or not at all, in some desiccation-tolerant organisms such as tardigrades and rotifers^14,18–21^. While this does not diminish the role of trehalose in mediating some instances of anhydrobiosis, it does point to the fact that other mediators must exist.

A recently emerging paradigm in the desiccation tolerance field is that anhydrobiosis can be mediated not only through the accumulation of massive levels of sugars, but also by the accumulation of intrinsically disordered proteins (IDPs)^14,15,22–29^. IDPs are proteins which lack stable three-dimensional structures, and instead exist in an ensemble of interconverting conformations^30–32^. IDPs are ubiquitous features of proteomes ranging from those of viruses to humans, and despite lacking stable three-dimensional structures play vital roles in many cellular and developmental phenomena^30–33^.

One IDP family that has recently garnered attention from the field of desiccation tolerance is the so-called Cytoplasmic Abundant Heat Soluble (CAHS) protein family^14,23,34–37^. CAHS proteins are unique to tardigrades, are required for these animals to robustly survive desiccation, increase desiccation tolerance when heterologously expressed in simple systems such as yeast and bacteria, and are sufficient to protect desiccation-sensitive enzymes during drying in vitro^14,23,35^.

Like many other anhydrobiotic organisms, tardigrades vitrify when dried, but seemingly only when expressing high levels of CAHS proteins^23,38,39^. In their purified state, CAHS proteins have been empirically demonstrated to form non-crystalline amorphous (vitrified) solids when dried, as have yeast heterologously expressing these proteins^23^. Vitrified CAHS proteins have been confirmed to plasticize with the addition of water, which is diagnostic, within the polymer field, of a vitrified material^39–41^. As mentioned above when discussing trehalose, vitrification is a phenomenon with a long-standing history in the desiccation tolerance field^7,12,38,42^, with proponents of the theory surmising that as an organism dries, the accumulation of vitrifying protectants serves to induce a super-viscous state in which detrimental effects of drying, such as protein unfolding and aggregation, are slowed to a point they do not take place on normal biological timescales. Consistent with this idea, disruption of the vitrified state of CAHS proteins, tardigrades, and other whole organisms has been shown to correlate with a loss of protective function^7,23,38^.

More recently, CAHS proteins have been implicated in the formation of hydrogels^34,36,37,43^, which often exist as non-crystalline, amorphous solids^44,45^. This lends further support to the notion that CAHS proteins form non-crystalline amorphous (vitrified) solids and that vitrification could be a mechanism underlying their protective capacity. It should be noted that vitrification, while necessary for anhydrobiosis, is not sufficient, meaning that vitrification must not be mutually exclusive with other possible mechanisms of protection^12^.

An alternative or additional mechanism that has been proposed for CAHS proteins is the mechanism of water retention^34,46^. The water retention hypothesis posits that a protectant, in this case a CAHS protein, could help protect an organism by retaining water, such that a dried tardigrade expressing CAHS proteins would contain more residual water than a dried tardigrade lacking CAHS proteins^9,24,47,48^. One of the main pieces of putative evidence that proponents of the water retention hypothesis point to is the fact that CAHS proteins form hydrogels^25,34,37^, with their reasoning being that some hydrogels retain a high level of water and therefore so too might CAHS hydrogels help to retain water when dried^34,46^. While there are bonafide examples of water retention serving as a protective mechanism^47,48^, to date, the water-retentive capacity of CAHS proteins in mediating stress tolerance remains in question.

Here we test the hypothesis that CAHS proteins mediate water retention. To assess whether or not CAHS proteins retain more water than common gelling and non-gelling proteins, as well as whether or not the gelled state of CAHS proteins influences their ability to retain water, we perform thermogravimetric analysis (TGA). TGA conducted on a model CAHS protein, CAHS D (Uniprot: P0CU50), in both a hydrated and dry state reveals that this protein retains no more water than common gelling and non-gelling proteins, as well as a variant of CAHS D which cannot gel. Furthermore, we assess the capacity of CAHS D to retain water in vivo and find that cells expressing CAHS D do not retain any more water than control cells, cells expressing a control protein (mVenus), or cells expressing variants of CAHS D which form stronger hydrogels or lack the ability to form gels.

However, we do find that water in CAHS D samples evaporates within an elevated range of temperatures relative to common proteins, indicating that while purified CAHS D protein or cells expressing this protein do not contain more water than control proteins or cells, the presence of CAHS D causes water to behave differently. This CAHS D-water interaction appears to be independent of the gelled state of CAHS D.

This study rules out water retention as a likely mechanism underlying CAHS-mediated desiccation tolerance. However, our results suggest that instead of retaining water, CAHS proteins interact with and influence the miniscule amounts of water left in dried systems, leaving open the possibility that CAHS-water interactions may underlie additional protective mechanisms.

Understanding the mechanisms by which tardigrades protect themselves and their biological macromolecules during desiccation advances our fundamental understanding of the phenomenon of anhydrobiosis. In addition, increased understanding of anhydrobiosis may provide potential avenues for pursuing real-world applications such as the preservation of pharmaceuticals in a dry, rather than cold, state and the generation of stress-tolerant crops and soil amendments for increasing food securing.

## Results

### CAHS D retains no more or no less water than common gelling and non-gelling proteins in vitro

To begin to assess whether water retention is a mechanism contributing to the protective capacity of CAHS proteins, and whether gelation of CAHS proteins specifically functions in water retention, we expressed and purified CAHS D (Uniprot: P0CU50), a model CAHS protein from the tardigrade *Hypsibius exemplaris*, which is known to form hydrogels and to provide protection during desiccation both in vitro and in vivo^14,22,23,34,36,37,43^. In addition, we purified an engineered variant of CAHS D termed CAHS D Full Length Proline (FL_Pro), which due to the insertion of three prolines in its carboxy-terminus lacks the ability to form hydrogels yet still provides protection to enzymes in vitro^36^. Gelling and non-gelling variants of CAHS D as well as two control proteins, gelatin (a gelling protein) and lysozyme (a non-gelling protein), which are not related to desiccation-tolerance, were tested using thermogravimetric analysis (TGA) both in their hydrated and desiccated states.

TGA is a widely used material science approach for determining the water content of a sample. Among other information, TGA provides a quantification of how much mass of a sample can be attributed to retained water (% water content) by heating the sample while simultaneously measuring its mass. As water evaporates a corresponding decrease in mass can be observed and a water content for the sample can be obtained. Additionally, this process allows one to measure the temperature at which water begins to be lost (onset), and the temperature at which all detectable water is lost (offset).

To begin, samples of gelatin (~ 100 kDa), lysozyme (14.3 kDa), CAHS D (25.6 kDa) and FL_Pro (25.6 kDa) were prepared at 8.7 mg/ml in 0.6 ml of milliQ water. At 8.7 mg/ml both CAHS D and gelatin form robust hydrogels, while FL_Pro and lysozyme do not^36^. Samples were kept in tubes sealed with parafilm to reduce evaporation and were loaded one at a time into a TGA crucible just prior to examination to reduce pre-testing evaporation. Here we use equivalent masses of protein rather than equimolar solutions to ensure that all samples begin with the same water:protein mass ratio.

**Figure 1:**
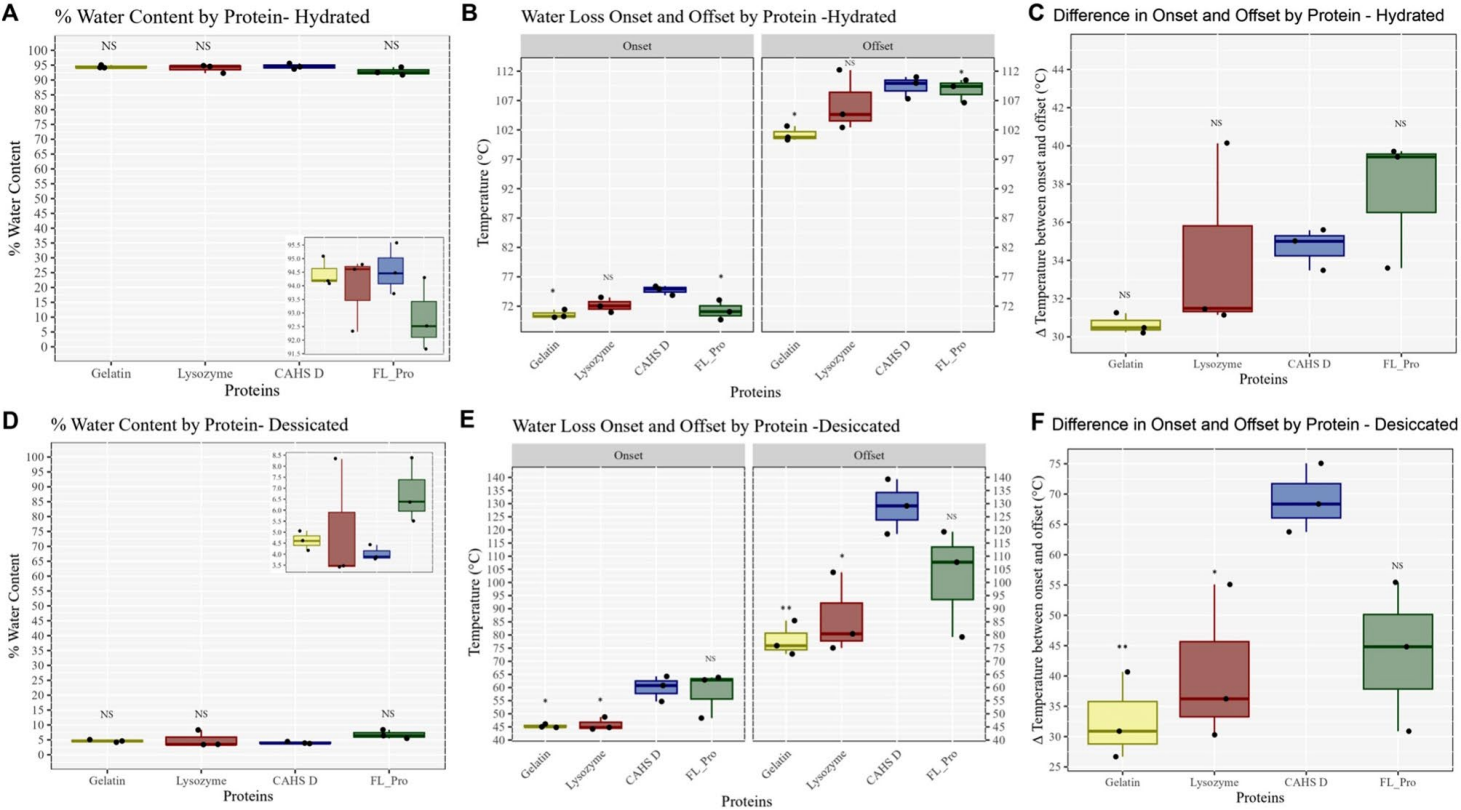
In vitro, CAHS D retains no more or less water than common proteins. (**A**) Quantitative water retention data for hydrated protein samples prepared at the same concentration. (**B**) Quantitative water-loss onset (temperature at which water begins to be lost) and offset (temperature at which all water has been lost) data for hydrated proteins. (**C**) Difference in onset and offset temperatures for hydrated proteins. (**D**) Quantitative water retention data for desiccated proteins dried side-by-side under the same conditions. (**E**) Quantitative water-loss onset and offset data for dried proteins. (**F**) Difference in onset and offset temperatures for desiccated proteins. Statistical significance was determined using one-way ANOVA and Tukey’s post-hoc test. Comparisons shown are to CAHS D. NS p > 0.05, *p < 0.05, **p < 0.01, ***p < 0.005.

As expected, solutions of all four proteins with the same concentration of protein in the same volume of water showed no statistical difference in water content (Fig. 1A). Of interest, CAHS D samples showed a modest, but statistically significant, increase in onset and offset temperature compared to gelatin and FL_Pro, but not to lysozyme (Fig. 1B). However, looking at the size of the temperature range at which water is lost (difference between onset and offset temperatures) we observed no difference between CAHS D and any of the three other proteins tested in a hydrated state (Fig. 1C).

These results indicate that in a hydrated state, water within mixtures of CAHS D, gelatin, lysozyme, and FL_Pro is not retained differently. CAHS D in a gelled state may interact with water more strongly compared to gelatin and non-gelling FL_Pro as indicated by its increased onset and offset temperature, but overall differences in water retention and interaction between CAHS D and the other proteins are not significant or modest at best.

Next, we prepared desiccated samples of gelatin, lysozyme, CAHS D, and FL_Pro and subjected these to TGA analysis to investigate whether CAHS D retains more water during desiccation. The average water content of dried CAHS D samples was 4.03%, which was not significantly different from the water content of dried gelatin (4.62%) or lysozyme (5.07%) (Fig. 1D). Interestingly, dried non-gelling FL_Pro retained significantly more water (6.77%, p < 0.05) than dried CAHS D (Fig. 1D).

The onset temperature at which water begins to be lost from dry CAHS D samples was observed to be significantly higher than gelatin and lysozyme. However, unlike in the hydrated state, the onset temperature for dried FL_Pro was similar to that of CAHS D (Fig. 1E). This trend was also observed for offset temperature, where CAHS D held onto water up to a higher temperature relative to gelatin and lysozyme, but not FL_Pro (Fig. 1E). This trend extends to the size of the range of temperatures over which CAHS D loses water, where a significant increase was observed for CAHS D compared to gelatin and lysozyme, but not FL_Pro (Fig. 1F).

Taken together these data indicate that in vitro CAHS D does not retain any more or any less water than common non-desiccation related gelling and non-gelling protein even during drying. Furthermore, the gelled state of CAHS D does not positively affect its water-retentive properties as the non-gelling FL_Pro retains more water than wild-type CAHS D when dried. Finally, while CAHS D does not retain more water than common proteins, it does appear to interact more tightly with the water molecules that are retained as evidenced by increased onset and offset temperatures.

### CAHS D retains no more or no less water regardless of gelation in cells

**Figure 2:**
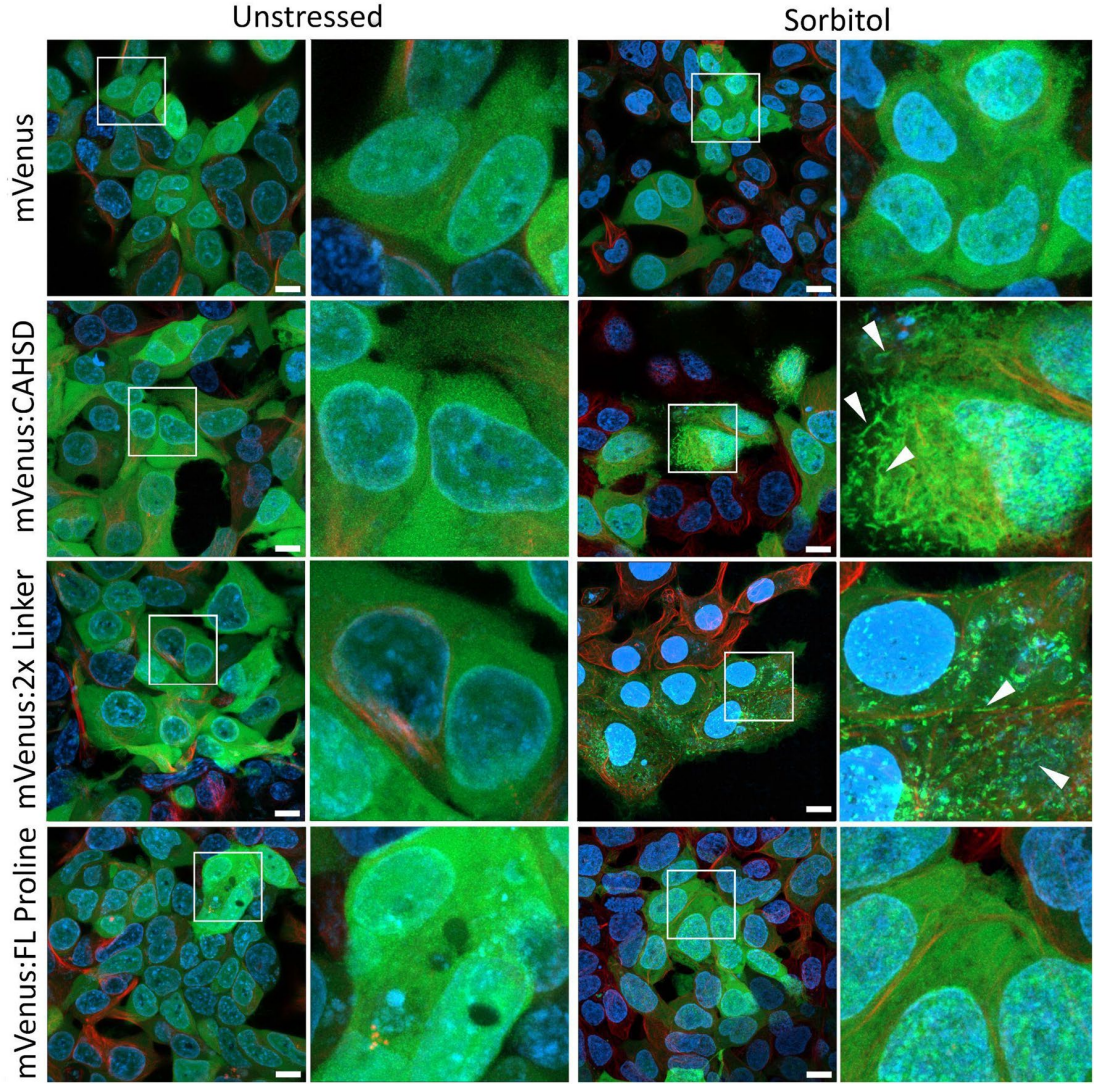
In vitro gelation of CAHS D and its variants is reflected in vivo by the appearance of fiber-like condensates upon osmotic stress. Maximum intensity projections, and inserts, of HEK cells stability expressing mVenus, mVenus:CAHS, mVenus:FL_Proline, or mVenus:2x Linker. Cells were either cultured and imaged under normal non-stressed conditions, or under osmotically stressed conditions (0.5 M Sorbitol for 4 h). The appearance of fiber-like condensation in mVenus:CAHS D and mVenus:2x Linker, but not mVenus:FL_Proline, under osmotic stress conditions mirrors the gelling properties (or lack thereof) of these proteins in vitro^36^. White arrows indicate fiber-like condensates observed in CAHS D and 2x Linker expressing cells upon osmotic shock. Blue = hoechst (DNA), red = SIR tubulin (microtubules), green = mVenus (monomeric mVenus or protein of interest). Scale bars = 10 µm.

Next, we sought to understand if CAHS D retains more water relative to a non-desiccation related protein, mVenus, in cells. To this end, stable lines of human embryonic kidney (HEK) cells were generated expressing mVenus, an N-terminal mVenus:CAHS D fusion, and CAHS D with a small C-terminal 1D4 tag (Fig. S1A,B) and were either left unperturbed or treated with sorbitol to induce osmotic shock. The 1D4 tag is an epitope from bovine rhodopsin. Here the 1D4 tag serves as a control to ensure that mVenus is not introducing artifacts. Additionally, sorbitol was selected to allow for comparisons to previous work carried out with CAHS proteins in cells^43,49^. CAHS proteins are known to form hydrogels in a concentration-dependent fashion in vitro and are suspected to do the same within cells during osmotic shock due to the observation of fiber-like formation and stiffening in vivo^25,34,43^. Consistent with this, sorbitol treatment resulted in the condensation of mVenus:CAHS D but not of mVenus alone (Fig. 2).

In addition, we sought to understand whether the condensed state of CAHS D influences its water retentive properties, since it was previously hypothesized that hydrogel formation of CAHS D would lead to water retention. To assess whether the condensed state of CAHS D influences its water-retentive properties, HEK cell lines stably expressing two variants of CAHS D were generated, mVenus:2x× Linker and mVenus:FL_Pro. As described above, FL_Pro does not form gels in vitro due to the insertion of 3 prolines in its C-terminus. Conversely, the 2x× Linker variant is the result of a tandem duplication of CAHS D’s internal linker region which results in a protein that forms gels at a lower concentration than wild type CAHS D in vitro^36^. Consistent with previous in vitro observations, mVenus:FL_Pro did not form condensates, while mVenus:2x× Linker does, in osmotically shocked cells (Fig. 2).

**Figure 3:**
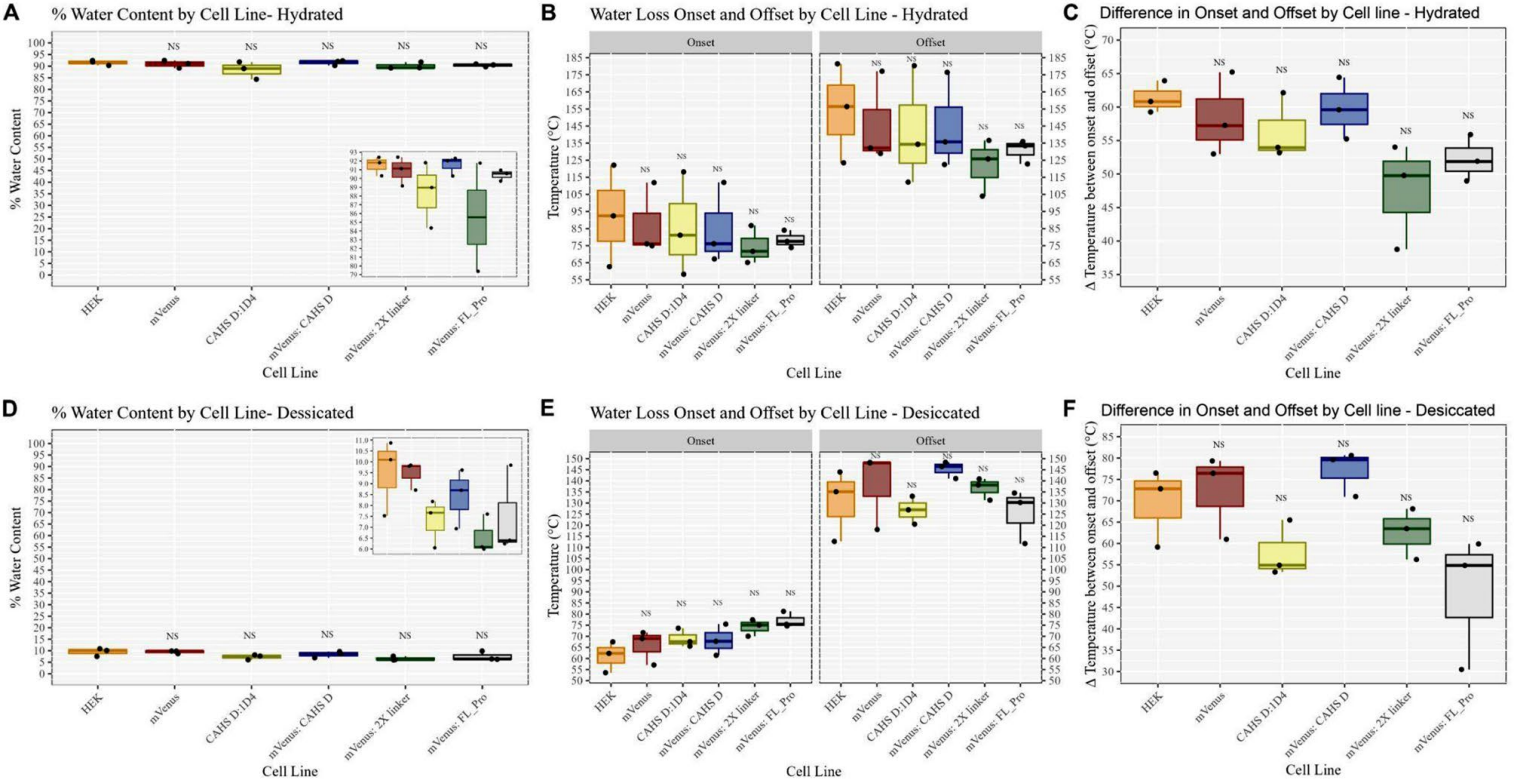
In vivo, CAHS D retains no more or less water than control cells or cells overexpressing a common protein. (**A**) Quantitative water retention data for hydrated cell lines. (**B**)  Quantitative water-loss onset (temperature at which water begins to be lost) and offset (temperature at which all water has been lost) data for hydrated cell lines. (**C**) Difference in onset and offset temperatures for each cell line used in this study in the hydrated state. (**D**) Quantitative water retention data for desiccated cell lines. (**E**) Quantitative water-loss onset and offset data for dried cells expressing mVenus, CAHS D:1D4, mVenus:CAHS D, mVenus:2x Linker, or mVenus:FL_Proline. (**F**) Difference in onset and offset temperatures for each cell line used in this study in the desiccated state. Statistical significance was determined using one-way ANOVA and Tukey’s post-hoc test. Comparisons shown are to control (HEK) cells. NS p > 0.05, *p < 0.05, **p < 0.01, ***p < 0.005.

TGA analysis of hydrated, non-osmotically shocked cells revealed that CAHS D:1D4 and mVenus:CAHS D expressing cells retain no more water, nor did they have a detectable increase in onset or offset for water loss, relative to control HEK cells or cells expressing mVenus (Fig. 3A).

Similar to control cells, hydrated non-osmotically shocked HEK cells expressing mVenus:FL_Pro or mVenus:2x Linker do not retain any more or any less water than CAHS D:1D4 or mVenus:CAHS D (Fig. 3A), nor did these cells have detectable differences in onset or offset (Fig. 3B) or did the difference in onset and offset vary between cell lines (Fig. 3C). Taken together, these data suggest that CAHS D does not alter water levels in hydrated cells.

Next, we reasoned that the proposed water-retentive properties of CAHS D might only manifest, or be detectable, at lower water content. To test this, we desiccated osmotically shocked control HEK cells and HEK cell lines stably expressing mVenus, CAHS D:1D4, mVenus:CAHS D, mVenus:2x Linker, or mVenus:FL_Pro and tested these dry cells via TGA (Fig. 3D,E). Osmotic shock was performed prior to drying to ensure that condensation took place before additional water loss, thus giving our test proteins the greatest chance of showing some difference in water retention.

Relative to control HEK cells, none of the cell lines tested retained any more or any less water (Fig. 3D). The onset and offset temperatures for water loss in control cells did not differ significantly from any other cell line, nor did the difference in onset and offset (Fig. 3E,F). Taken together, these results demonstrate that CAHS D does not contribute to water retention in cells.

## Discussion

The discovery that CAHS proteins help to mediate desiccation tolerance in tardigrades^23^ and form hydrogels^34,36,37^ has led to several studies aimed at identifying the mechanism or mechanisms by which CAHS D acts to promote desiccation tolerance and whether these mechanisms are linked to gelation^34,36^.

Water retention, in which CAHS proteins might help increase the total residual water left in the dry system, has been proposed as a potential mechanism by which CAHS protein may function as a protectant^34,46^. This speculation has formed largely around the thought that hydrogels contain water and therefore might help to retain more water during desiccation^34^.

To directly test this theory, we measured the amount of water retained by CAHS D in a gelled/condensed and non-gelled/uncondensed state, both in vitro and in cells via TGA. We find that CAHS D retained no more and no less water than common non-desiccation-related proteins or control cells not expressing CAHS D. The singular observation of increased water retention in our study was for the non-gelling variant of CAHS D, FL_Pro, which in vitro retained ~ 6.77% water when dried relative to CAHS D which retained ~ 4.03% water when dried (p < 0.05). Thus, if anything, our study suggests that gelation of CAHS D may be antagonistic to water retention.

Here, we have tested purified proteins in vitro or proteins expressed in cells, but in non-tardigrade cells, leaving open the possibility that the water-retentive properties of CAHS proteins might differ in tardigrades. It should be noted that to date all studies on the gelation of CAHS proteins has been carried out in vitro or in heterologous ex vivo systems. Thus, our study is in line with norms within the field for assessing putative mechanisms of protection.

Reverse genetics could serve as an approach to test the mechanistic underpinnings of anhydrobiosis in tardigrades. However, to date RNAi remains the only fully developed methodology of reverse genetics in tardigrades. This methodology requires microinjection of individual animals and given the large amount of sample input (~ 10 mg) required for TGA is impractical in this case. However, TGA analysis on non-conditioned tardigrades, which express CAHS genes at relatively low levels, versus conditioned tardigrades, which express CAHS proteins at high levels, have been performed and shows that dried non-conditioned tardigrades retain more water than conditioned specimens^39,46^. This study provides good direct evidence that anhydrobiotic tardigrades do not retain more water than non-anhydrobiotic tardigrades, and good indirect evidence that CAHS proteins do not participate in water retention within these animals themselves.

Based on direct empirical evidence (TGA studies on purified protein and cells) and indirect evidence (TGA studies on conditions versus non-conditioned tardigrades), we conclude that hydrated or dry systems containing CAHS proteins do not contain more water than hydrated or dry systems lacking CAHS proteins, and thus water retention is likely not a mechanism underlying their desiccation-protective properties.

In addition to measuring the total amount of water in a system, TGA provides insights into the temperature at which water begins to leave (onset) and has detectably fully left a system (offset). Such information can indicate something about the state of water within a system, for example whether it is behaving like free liquid water or is interacting with other components of the system.

Of interest is the observation that in vitro dry samples of CAHS D and its non-gelling variant FL_Pro have elevated onset and offset temperatures relative to control gelling and non-gelling proteins (Fig. 1E). This indicates that while CAHS D does not retain more water in the dry state, it does interact with water in the dry state more tightly. The observation that the non-gelling variant FL_Pro’s onset and offset temperatures were not significantly different from that of gelling CAHS D’s (Fig. 1E) indicates that this interaction with water is not governed by the gelation state of CAHS D.

It should be noted that increased onset/offsets were only observed in dry purified samples. This could be due to several possibilities. First, in hydrated samples vastly more water–water interactions exist than do water–CAHS interactions. TGA may not have the sensitivity to detect these relatively rare water–CAHS interactions under such conditions. However, in the dry state the ratio of water–water to water–CAHS interactions is greatly shifted towards the latter, which TGA now has the power to detect. Secondly, CAHS proteins are known to undergo a structural shift during drying/desolvation^25,35,36^, going from a largely disordered state to a state with increased helical content. Bioinformatic analysis indicates that the helices that form upon drying in CAHS proteins are strongly amphipathic^25,35,36^. The rearrangement of hydrophilic residues to one of the faces of this helix could serve to strengthen water–CAHS interactions.

Another interesting feature of CAHS gels is that they have been observed to readily go back into solution both in vitro and in vivo^34,36^. This lends further credence to the idea that CAHS proteins readily interact with water (as evidenced by increased onset temperatures), but does not imply anything about the proteins water retentive capacity.

This study does not rule out the possibility that gelation of CAHS proteins can be mechanistically linked to tardigrade anhydrobiosis, but it is clear that gelation of these proteins is not playing a role in water retention. The formation of a gelled matrix of protein may serve as a desiccation inducible cytoskeleton that helps to maintain the organization and ultrastructure of drying cells, such that during drying membranes do not collapse and fuse.

Interestingly, while CAHS D has been observed to undergo a phase transition, going from a solution to gelled state, we did not observe this protein to undergo phase separation. This is not the case for other CAHS proteins, or for some members of another group of desiccation related IDPs known as late embryogenesis abundant (LEA) proteins, that have been reported to form liquid–liquid phase separation^43,50^. Liquid–liquid phase separation of desiccation-related IDPs could serve to promote desiccation tolerance by sequestering and protecting vital proteins and other cellular components or by setting up regions with chemistries, biophysical, or material properties that promote protection^43,50^. In the future it will be important to compare and contrast the function consequences of a desiccation-related IDP’s phase transition versus phase separation on protective capacity during drying.

While further work will be needed to test whether the interaction between CAHS proteins and water is a mechanism underlying the protective capacity of these proteins during water deficit, one can envision that CAHS proteins might act as water aggregators, concentrating but not increasing the minuscule amounts of residual water left in dried tardigrades. This could help maintain local areas of hydration, which in turn could help to preserve the structure, integrity, and function of essential labile biomolecules. This idea is contrary to the typical mantra that anhydrobiotic organisms are ametabolic in their dry state, however the claim that dried tardigrades and other organisms lack any metabolism has recently been challenged. For example, studies in desiccated yeast show that trehalose degrades over time and that this degradation is dependent on the presence of trehalase, an enzyme required for the breakdown of trehalose^51^. It is important to note that in this study desiccation of yeast was carried out at 60% relative humidity at 23 °C, which is likely insufficient to achieve the commonly recognized level of water in anhydrobiotic organisms (< 0.1 g of water per gram of dry mass), which typically requires drying at 50% relative humidity at 20 °C^52^.

Other mechanisms might also underlie CAHS proteins’ protective capacity, such as vitrification or the formation of non-crystalline amorphous solids^23,38,39^. While vitrification has previously been empirically measured in tardigrades and for CAHS proteins^23,38^ and confirmed via plasticization assays^39^, the more recent observations that CAHS proteins form gels lend credence to the notion that CAHS proteins undergo vitrification, as gels themselves are often non-crystalline amorphous solids^34,36,37,44,45^.

It should also be noted that while the vitrification hypothesis is not mutually exclusive with many other putative mechanisms of desiccation tolerance, it is at odds with the water retention hypothesis. This is because water is known to be a strong plasticizer of vitrified materials^53,54^, including vitrified CAHS protein^39^, and plasticization of dried systems has been linked to loss of protective capacity^54,55^.

Taken together, our study suggests that while there is still much to learn about the mechanism(s) underlying tardigrade desiccation tolerance, in the hydrated and dry state water retention is not a measurable property attributable to CAHS proteins in a gelled or non-gelled state. Furthermore, our study suggests that while CAHS D does not retain more water in the dry state, it does appear to interact with and influence the properties of water within some systems, indicating that tighter protein-water interactions may be a mechanism underlying the protective capacity of CAHS proteins.

## Methods

### Obtaining proteins used in this study

Gelatin and Lysozyme were purchased from Sigma: Cat: G1890-100G and Cat: L6876-5G, respectively. CAHS D (Uniprot: P0CU50) and FL_Pro were expressed and purified in house using established protocols^36^.

### Protein expression and purifications

CAHS D and FL_Pro proteins were expressed and purified using established protocols^36^. Briefly, pET28b plasmids containing a codon-optimized gene encoding the protein of interest were transformed into BL21 bacteria. Following outgrowth and plating, a single colony was grown overnight in liquid Luria Broth supplemented with Kanamycin (50 μg/ml). Overnight cultures were used to inoculate 1 L Luria Broth cultures supplemented with Kanamycin (50 μg/ml). Cultures were grown at 37 °C until an optical density of 0.6 was reached. Dense cultures were then induced with 1 mM IPTG and grown for an additional 4 h. After expression, cells were harvested by centrifugation at 3500 rpm for 30 min. The supernatant was discarded, and cells were resuspended in 5 ml of pellet resuspension buffer (20 mM Tris pH 7.5) supplemented with protease inhibitors. Pellets were stored at − 80 °C until use.

Pellets were thawed at room temperature and subjected to heat lysis in boiling water for 10 min and allowed to cool to room temperature. Boiled samples were then centrifuged at 10,500 rpm for 45 min at 10 °C, and the supernatant was filter-sterilized through a 0.22 μm syringe filter (EZFlow Syringe Filter, Cat. 388-3416-OEM) to remove any insoluble particles. The filtrate was diluted two times its volume with buffer UA (8 M Urea, 50 mM sodium acetate, pH 4). Diluted lysates were loaded onto a HiPrep SP HP 16/10 cation exchange column (Cytiva) and purified on an AKTA Pure, controlled using the UNICORN 7 Workstation pure-BP-exp.

CAHS D and FL_Pro were eluted using 70% UB gradient (8 M Urea, 50 mM sodium acetate, and 1 M NaCl, pH 4) and fractionated over 15 column volumes.

Purified protein fractions were confirmed using SDS-PAGE and selected fractions were pooled for dialysis in a 3.5 kDa tubing in 20 mM sodium phosphate buffer pH 7. This was followed by six rounds of dialysis in Milli-Q water (18.2 MΩcm) at 4 h intervals each. Samples were quantified fluorometrically using Qubit 4 fluorometer, flash frozen, then lyophilized for 48 h (Labconco FreeZone 6, Cat. 7752021) and stored at − 20 °C until further use.

### Preparation of hydrated protein samples

Hydrated protein samples were prepared via resuspension of proteins at 8.7 mg/ml in Milli-Q water (18.2 MΩcm). Samples were heated at 55 °C for 15 min and visually inspected to ensure full solvation. Samples were then subjected to TGA analysis one at a time to avoid evaporation in TGA crucibles (Cat. T221108, TA Instruments) while analysis runs. If not being tested on the TGA, samples were kept in tubes sealed with parafilm to further reduce the risk of evaporative loss. Stored samples were always tested within 4 h of preparation. All stored samples were briefly heated for 5 min at 55 °C prior to loading on TGA pans since gelling proteins (CAHS D and gelatin) require this for ease of handling.

### Preparation of desiccated protein samples

Protein samples were prepared at 8.7 mg/ml as indicated above. Samples were transferred to 1.5 ml Eppendorf tubes and desiccated for 16 h in a vacuum concentrator (Savant SpeedVac Vacuum Concentrator Model SC110-120). After desiccation samples were stored in tubes sealed with parafilm in a glass desiccating chamber filled with Indicating Drierite (Cat. 23005). Just prior to TGA analysis, samples were removed from tubes and manually broken into a powder-like state on a weight boat with a spatula to ensure homogenization of the sample.

### Generation of stable cell lines

Full-length CAHS D (Uniprot P0CU50, CAHS 94063), CAHS D variants FL_proline and 2x Linker with mVenus protein fused in their N-termini, mVenus and Full-length CAHS D (Uniprot P0CU50, CAHS 94063) with 1D4 epitope fused in its C-termini were cloned into pTwist-cmv-WPRE-Neo between HindIII and BamHI and sequence verified (TwistBioscience Inc.). 1 µg of plasmid DNAs expressing CAHS D:1D4, mVenus, mVenus:CAHS D, mVenus:FL_ proline and mVenus:2x Linker proteins were transfected into Hek293 cells (Cat. CRL-1573, ATCC) with lipofectamine 3000 transfection reagent (Cat. L3000008, Thermo Fisher Scientific). 24 h post-transfection, cells that had successfully integrated CAHS D:1D4, mVenus, mVenus:CAHS D, mVenus:FL_proline and mVenus-2x Linker were selected with 0.7 µg/µl G418 (Cat. G64500.20.0, Research products international). Cell lines were passed twice before expanding and flash-cooling. Stable cell lines were maintained supplementing Dulbecco’s modified Eagle’s medium (DMEM) (Cat. 10567014, Gibco) media with 10% fetal bovine serum (FBS), (Cat. 900-108, GeminiBio Products), 1% penicillin/streptomycin (Cat. 400-109, Gemini Bio products) and 0.3 µg/µl G418 (Cat. G64500.20.0, Research products international) at 37 °C with 5% CO_2_ atmosphere.

### Fluorescence imaging of cell lines

8-well glass bottom dishes (Cat. 80826, Ibidi) were pre-coated with 0.1 mg/ml poly-d lysine (Cat. A3890401, Thermo Fisher Scientific) for 1 h at 37 °C. Cells expressing mVenus, mVenus:CAHS D, mVenus:FL_Proline mVenus:2x Linker and CAHS D:1D4 were seeded in duplicate at a density of 2.5 × 10^5^ cells/ml and allowed to recover overnight. After one day, one set of the cells were treated with 0.5 M sorbitol in growth media for 4 h while the other set was fluid changed in growth media. After the 4 h incubation the medium from both sets was replaced with an imaging medium (FluoroBrite DMEM, Cat. A1896701, Thermo Fisher Scientific) supplemented with 0.5 µg/ml Hoechst 33342 dye (Cat. 62249, Thermo Fisher Scientific) and 1 µM SIR Tubulin dye/10 µM verapamil (Cat. CY-SC006, Cytoskeleton inc.) for the mVenus fusion proteins and with 0.5 µg/ml Hoechst 33342 dye (Cat. 62249, Thermo Fisher Scientific) for CAHS D: 1D4, then they were incubated for 30 min with the dyes prior imaging.

Images were acquired using a Zeiss 980 Laser Scanning Confocal microscope equipped with a Plan-Apochromat 63 × oil objective, 40 × multi-immersion LD LCI Plan-Apochromat objective and a 20x air Plan-Apochromat objective (Zeiss Instruments). Data acquisition used ZEN 3.1 Blue software (Zeiss Instruments). Hoechst 33342 dye (Cat. 62249, Thermo Fisher Scientific) was excited by 405 nm laser light and the spectral detector set to 409–481 nm. mVenus protein was excited by 488 nm laser light and the spectral detector set to 490–550 nm. SIR Tubulin dye (Cat. CY-SC006, Cytoskeleton inc.) was excited with 639 nm laser light and the spectral detector set to 640–720 nm, CAHS D:1D4 protein was excited by 561 nm laser and the spectral detector set to 573–627 nm. Images were processed using ZEN 3.1 Blue software airyscan tool. Data analysis was performed in fiji.

### Preparation of hydrated cell samples

Hydrated and desiccated cells were seeded, treated and collected in parallel the same days.

Cells expressing CAHS D:1D4, mVenus, mVenus:CAHS D, mVenus:FL_Proline and mVenus:2x Linker were each seeded in three T-75 flasks (Cat. SP81186, Bio-Basic) at a density of 1.0 × 10^6^ cells/ml and grown until confluency. Once the cells reached confluency, the media was changed and cells were trypsinized and collected by centrifugation at 100×*g* for 10 min, media was removed by aspiration and cell pellets were transferred directly into TGA crucibles (Cat. T221108, TA Instruments) that had been pre-massed on TA Instruments TGA5500 device using TA Instruments Trios software (v5.5.0.232).

### Preparation of desiccated cell samples

Cells expressing mVenus, mVenus:CAHS D, mVenus:FL_Proline and mVenus 2x Linker were each seeded in three T-75 flasks (Cat. SP81186, Bio-Basic) at a density of 1.0 × 10^6^ cells/ml and grow until confluency. Once the cells reached confluency, the three T-75 flasks were treated with 0.5 M sorbitol in growth media for 4 h. After the sorbitol incubation the cells were trypsinized and collected by centrifugation at 100×*g* for 10 min, media was removed by aspiration and cell pellets were transferred directly into TGA crucibles (Cat. T221108) that had been pre-massed on TA Instruments TGA5500 device using TA Instruments Trios software (v5.5.0.232). Crucibles with samples were transferred to a sealed glass desiccating chamber filled with Indicating Drierite (Cat. 23005) for 16 h.

### Thermogravimetric analysis (determination of % water, onset, and offset)

Samples were loaded into TA instrument TGA crucibles (Cat T221108). TGA was conducted using a TA Instruments TGA5500 device using TA Instruments Trios (v5.5.0.232). For all samples, our TGA protocol consisted of an equilibration at 30 °C, followed by a 10 °C/min ramp to 200 °C. Percent water content, onset, and offset temperatures were determined using TA Instruments Trios software (v5,5,0.232) Intelligent, Onset, and Endset tools, respectively.

### Western blot

Naive Hek 293 cells and cells expressing CAHS D:1D4 were each seeded in a T-75 flasks (Cat. SP81186, Bio-Basic) at a density of 1.0 × 10^6^ cells/ml and grown until confluency. Once the cells reached confluency, the media was changed and cells were trypsinized and collected by centrifugation at 100×*g* for 10 min, media was removed by aspiration and cell pellets were resuspended in 1 ml 20 mM Tris–HCl, pH 7.4. 100 μl of the cell lysates were set aside and mixed with 2x Laemmli sample buffer (cell lysate sample). 900 μl of the cell lysates were heat solubilized by boiling them for 10 min. After heat solubilization samples were centrifuged at 13,000×*g* for 30 min to separate the soluble components from the insoluble components. The supernatants were transferred to clean Eppendorf tubes and 100 μl of each were set aside and mixed with 2x Laemmli sample buffer (supernatant sample). The insoluble fractions were resuspended in 900 μl of mM Tris–HCl, pH 7.4 buffer, 100 μl were set aside and mixed with 2x Laemmli sample buffer (pellet samples).

10 μl of samples were loaded in a 4–20% Criterion TGX Precast protein gel (catalog; 5671094, Bio-Rad) and separated by running the gel at 150 V for 45 min. Precision Plus Protein Dual Xtra prestained protein Ladder (Catalog 1610377, Bio-Rad) was used as the size standards. Samples were transferred onto a polyvinylidene difluoride membrane activated with methanol using the Trans-Blot Turbo Transfer System (Bio-Rad). Membranes were probed with mouse Rho-1D4 antibody (Catalog 40020, Cube Biotech) diluted 1:3000 in Western Breeze Chromogenic Immunodetection kit's (Catalog WB7103, Thermo Fisher Scientific) primary antibody diluent. For Rho-1D4 detection Western Breeze Chromogenic Immunodetection kit instructions were followed.

### Statistical analysis of data

Data was compiled in Microsoft Excel and analyzed using R. For all figures presented one-way Analysis of Variance (ANOVA) tests were used to determine significance. Further analysis using Tukey’s post-hoc tests were used to determine statistical differences between experimental groups.

## Supplementary Information

Supplementary Information 1.

Supplementary Information 2.

Supplementary Figure 1.

## Data Availability

All raw data and analyzed plots used here are provided in File S1.zip. All scripts and code used here are provided in File S2.zip.
