# Supplementary material for: The tardigrade protein CAHS D interacts with, but does not retain, water in hydrated and desiccated systems
Source: Sci Rep. 2023 Jun 27;13:10449. doi: 10.1038/s41598-023-37485-3 (PMC10300006; doi:10.1038/s41598-023-37485-3)
Supplement: Supplementary file 3 — Supplementary Figure 1. [file 41598_2023_37485_MOESM3_ESM.docx]

Supplemental material for Sanchez Martinez et al.,

Supplemental Figures


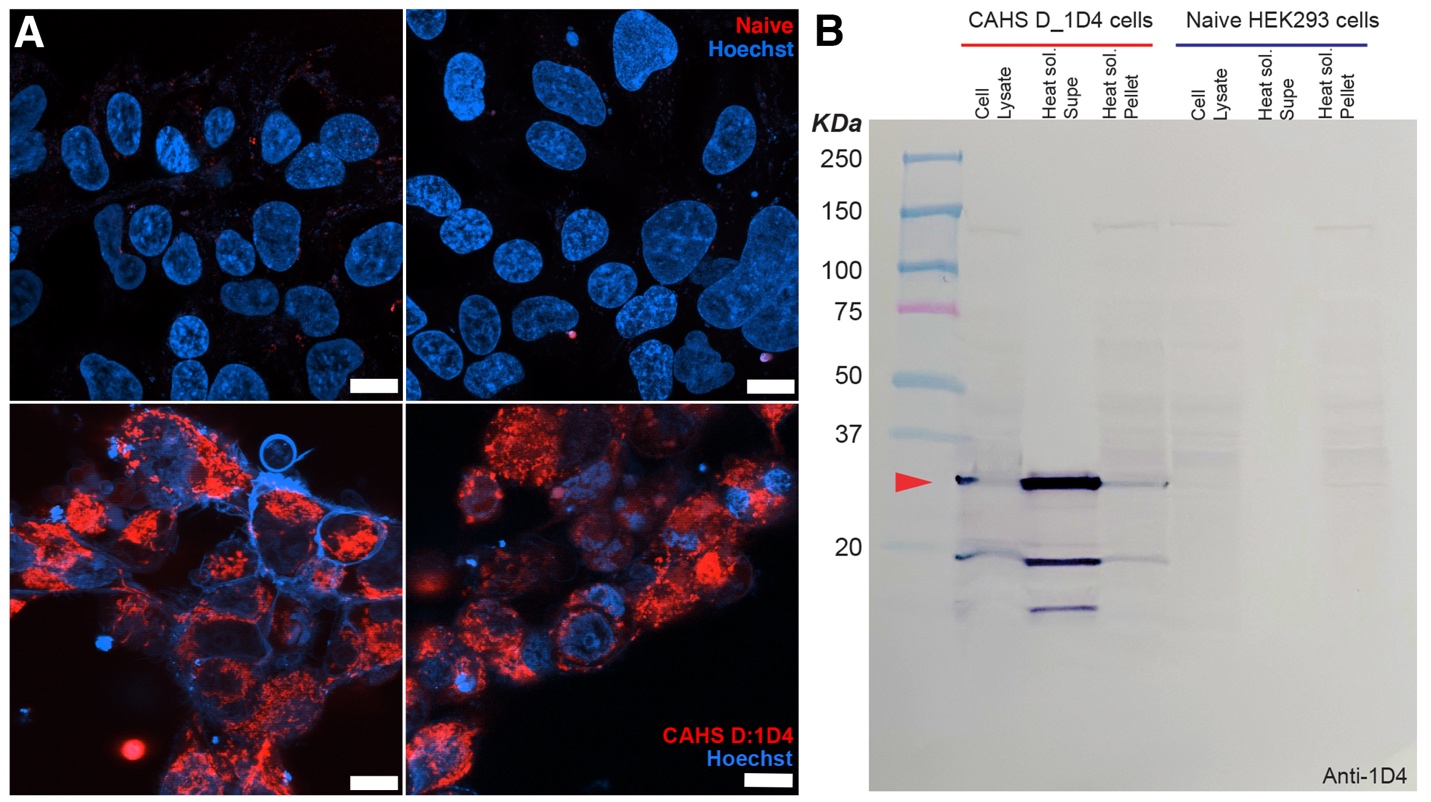


Supplemental Figure 1 – Expression of CAHS D:1D4 in osmotically shocked cells. A) Representative images of naïve (top) and CAHS D:1D4 (bottom - red) expressing HEK cells. Cells were osmotically shocked with 0.5M sorbitol. Cells counterstained with Hoechst (blue). Scale bar = 10 um. B) Western blot using an anti-1D4 antibody.
